# Sphincter of Oddi Function and Risk Factors for Dysfunction

**DOI:** 10.3389/fnut.2017.00001

**Published:** 2017-01-30

**Authors:** Elham Afghani, Simon K. Lo, Paul S. Covington, Brooks D. Cash, Stephen J. Pandol

**Affiliations:** ^1^Cedars-Sinai Medical Center, Los Angeles, CA, USA; ^2^Clinical Dynamix, Wilmington, NC, USA; ^3^University of South Alabama, Mobile, AL, USA

**Keywords:** pancreatitis, sphincter of Oddi, sphincter of Oddi dysfunction, functional biliary disorder, biliary colic, hepatic enzymes, lipase, amylase

## Abstract

The sphincter of Oddi (SO) is a smooth muscle valve regulating the flow of biliary and pancreatic secretions into the duodenum, initially described in 1887 by the Italian anatomist, Ruggero Oddi. SO dysfunction (SOD) is a broad term referring to numerous biliary, pancreatic, and hepatic disorders resulting from spasms, strictures, and relaxation of this valve at inappropriate times. This review brings attention to various factors that may increase the risk of SOD, including but not limited to: cholecystectomy, opiates, and alcohol. Lack of proper recognition and treatment of SOD may be associated with clinical events, including pancreatitis and biliary symptoms with hepatic enzyme elevation. Pharmacologic and non-pharmacologic approaches are discussed to help recognize, prevent, and treat SOD. Future studies are needed to assess the treatment benefit of agents such as calcium-channel blockers, glyceryl trinitrate, or tricyclic antidepressants in patients with SOD.

## Historical Notes and Purpose of the Review

In 1941, Williamson wrote a letter to the British Medical Journal, concerned that “a fair number of people without gallbladders who are potential air raid causalities and to whom … morphine will probably be administered … (would), far from relieving their pain and shock … increase it” (1). The letter was in response to a publication by Smyth, who noted that selected drugs dilate the sphincter of Oddi (SO) while, “contrary to what might be expected,” morphine, codeine, and hydromorphone produced “spasm of the sphincter” (2). This formed the basis for the syndrome later termed SO dysfunction (SOD). The purpose of this paper is to review SO anatomy and physiology, SOD (including subtypes), the effect of cholecystectomy on the SO, and the impact of exogenous compounds on the SO, and to provide an overview of the diagnosis and management of SOD.

## Structure and Function of the SO

**Figure 1:**
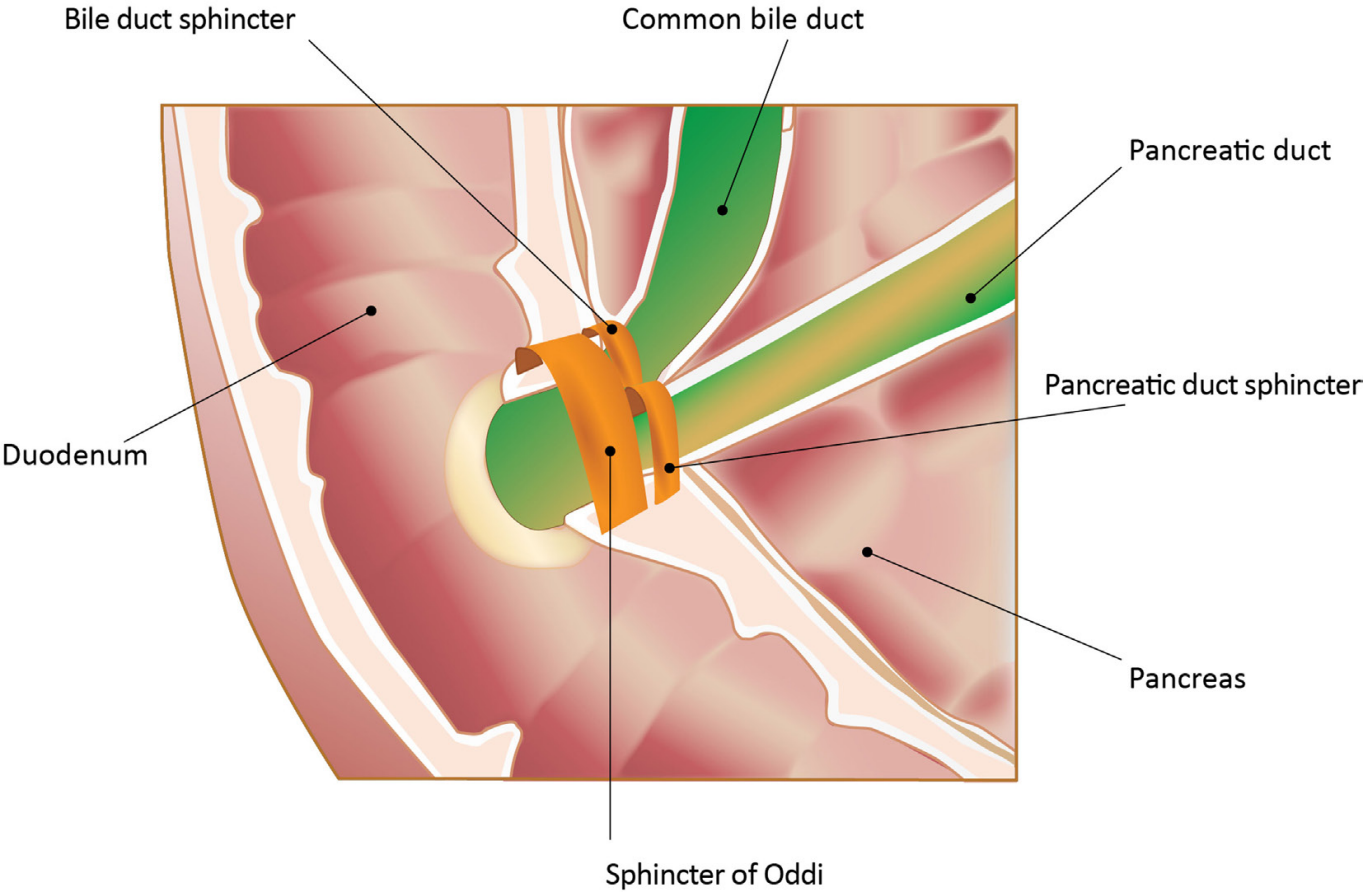
**Sphincter of Oddi (SO) and its anatomic relationships**. In the majority of patients, pancreatic and biliary secretions enter the duodenum through the SO. The sphincter structure with overlying mucosa protruding into the duodenum is called the papilla of Vater. The image is an endoscopic photograph of the duodenum at this entry site. In addition, there are individual sphincters for both the common bile duct and the pancreatic duct proximal to the SO. All the sphincters are neurohumorally regulated. Adapted from Gorelick et al. (5). Used with permission. Copyright, American Gastroenterological Association Institute, Bethesda, MD, USA.

The SO is a muscular structure surrounding the confluence of the distal common bile duct (CBD) and the pancreatic duct (PD) into the ampulla of Vater (Figure 1) (3, 4). The sphincter structure with overlying mucosa protruding into the duodenum is called the papilla of Vater. At least three functions of the SO have been identified: (1) regulation of bile flow into the duodenum; (2) prevention of duodenal reflux; and (3) regulation of gallbladder filling by diverting bile into the gallbladder with SO closure.

The physiology of the SO has been studied in both animals and humans. While varying species have been studied, dog, cat, and pig physiology are most similar to humans. It is critical to consider species studied when analyzing pharmacological data (see Medications and the Risk of SOD). In humans, basal pressure of the SO is 10 mmHg. Superimposed anterograde phasic contractions, initiated at the junction of the CBD and the SO and progressing into the duodenum, occur in response to physiologic and exogenous stimuli and result in evacuation of contents already present within the SO into the duodenum. During contraction, no additional flow from the CBD into the SO occurs. The SO then relaxes, allowing passive refilling of bile into the SO segment. Once filled, another wave of phasic contractions begins (6, 7). When basal pressure increases, resistance to flow increases, resulting in gallbladder filling and prevention of flow into the duodenum. When basal pressure decreases below CBD and PD pressures, flow into the duodenum occurs.

Sphincter of Oddi motility patterns have been defined for both the inter-digestive period and the digestive period. During the inter-digestive period, the SO has a cyclical activity pattern throughout all phases and the frequency increases prior to phase 3 duodenal activity, prompting discharge of bile and pancreatic secretions into the duodenum (8–11). Motility during the digestive period involves both neural and hormonal inputs. In this period, there are gallbladder contractions, stimulation of pancreatic secretion, and SO relaxation, leading to high rates of bile and pancreatic secretion into the duodenum. Of note, during the digestive period, there are different phases of the meal, each providing input. For example, up to 30–40% of gallbladder emptying and 25% of pancreatic secretion occurs during the cephalic phase *via* vagal inputs, while another 10–20% of the response occurs during the gastric phase *via* vagovagal pathways. However, the gallbladder empties most of its remaining contents and the pancreas up to 50% of its total secretion during the intestinal phase, induced by the release of cholecystokinin (CCK) and secretin from the duodenum and proximal jejunum. Duodenal CCK contracts the gallbladder, relaxes the SO, and causes pancreatic exocrine digestive enzyme secretion *via* direct actions on CCK receptors and indirectly through cholinergic neurons. This is supported by the observation that atropine pretreatment blocks gallbladder contraction and pancreatic secretion induced by physiological concentrations of CCK and by a protein-fatty meal (12–15).

## Hormones and Neurotransmitters Involved in SO Function

The most important hormone involved in SO function is CCK. CCK released from the enteroendocrine cells in response to a meal exerts direct hormonal effects as well as indirect effects by interacting with neural pathways, leading to gallbladder contraction and pancreatic enzyme secretion. CCK decreases SO basal pressures and inhibits phasic contractions, thereby promoting anterograde flow (16, 17). Vasoactive intestinal polypeptide and nitric oxide, present in the intrinsic neurons of the SO, are involved in the relaxation response to CCK as well as the relaxation observed in the cephalic phase of the meal (18, 19).

Motilin, somatostatin, and octreotide hormonally influence SO function. Motilin, secreted by the M cells within the duodenum and jejunum, induces contraction of the smooth muscle of the gallbladder and stimulates bile secretion (20–22). Somatostatin, present in endocrine cells throughout the gastrointestinal tract, likely exerts inhibitory effects on both gallbladder contraction and relaxation of the SO (23), supported by the observation that 50 μg intravenous octreotide, an agent that mimics somatostatin, causes a significant increase in SO basal pressure and frequency of phasic contractions (24).

## What is SOD?

Sphincter of Oddi dysfunction is a clinical syndrome caused by SO dyskinesia (functional) or anatomic (mechanical) obstruction associated with abdominal pain and elevation of liver or pancreatic enzymes, CBD or PD dilation, or pancreatitis (25). Of note, the term SO dyskinesia more specifically denotes motility disorders of the SO, while SOD includes both mechanical obstruction and SO dyskinesia. In this context, the term biliary dyskinesia has historically been used as a general term that included both SO dyskinesia and gallbladder dyskinesia (26). Since the availability of scintigraphy, a functional gallbladder disorder (i.e., gallbladder dyskinesia/dysfunction) is now recognized as a discrete entity and should be distinguished from SO dyskinesia (27–29). The various forms of primary SOD are considered functional gastrointestinal disorders and may occur in adults or children of any age, but SOD is most commonly encountered in females aged 20–50 years (30–32). The estimated prevalence of SOD is 1.5% in the general population and may be as high as 72% in patients with idiopathic recurrent pancreatitis based on small cohort studies (30, 32–35). However, its true prevalence is difficult to determine due to the lack of definitive biomarkers or diagnostic criteria as well as the multitude of secondary causes of SOD, such as fibrosis of the sphincteric channel (papillary stenosis and sclerosing papillitis) or obstructive carcinoma.

## Clinical Presentations of SOD

Sphincter of Oddi dysfunction can involve the biliary sphincter, the pancreatic sphincter, or both (25). Biliary SOD typically presents with recurrent biliary pain, characterized as disabling epigastric or right upper quadrant pain lasting 30 min to several hours with or without hepatic enzyme elevation. It may radiate to the back, shoulder, or scapula and may be accompanied by nausea and vomiting, mimicking a gallbladder attack. Pain is not consistently postprandial and is not relieved by postural changes, antacids, or bowel movements.

Pancreatic SOD is thought to be responsible for a portion of patients with recurrent episodes of acute pancreatitis. Patients will have mid-abdominal, pancreatic pain, radiating to the back, associated with elevations in serum amylase and lipase. Symptoms involving the pancreatic sphincter are frequently exacerbated by food intake. No other causes for pancreatitis are usually found in these patients, and they may be classified as having idiopathic acute recurrent pancreatitis (IARP) (36, 37). Toouli and colleagues compared the SO manometric pressures in 28 patients with IARP with those of 10 controls and found that more than 57% of patients with IARP had elevated SO pressures (38). However, the true incidence of pancreatitis caused by SOD is unknown. When both the pancreas and biliary sphincters are involved, the abdominal pain may be more diffuse and both hepatic and pancreatic enzyme elevation can occur.

## Diagnosis of SOD

The diagnosis of SOD is challenging, but history, physical exam, relevant labs, and imaging studies are critical. Some view SOD as a structural abnormality while others view it as a functional disorder. A classification system for SOD as a structural abnormality was established in 1988. Initially known as the Hogan–Geenan SOD classification and later modified as the Milwaukee classification, it classifies SOD patients into types 1, 2, and 3 based on clinical presentation as well as laboratory and/or imaging abnormalities (see Table 1 for biliary SOD and Table 2 for pancreatic SOD) (26, 39). Table 3 lists the Rome III criteria for SOD as a functional disorder (25). These criteria were meant to make the diagnostic evaluation more applicable to clinical practice and, whenever possible, avoid invasive procedures by emphasizing non-invasive imaging of CBD diameter. Earlier studies showed higher rates of depression, obsessive compulsive disorders, and anxiety in patients with type 3 SOD when compared with controls (40). Conversely, a randomized, controlled trial of SOD type 3 patients showed that psychosocial disability in patients with severe symptoms may not be different than in the general population (41).

**Table 1:** **Milwaukee classification for biliary SOD (39)**.

**Type 1**	Biliary pain associated with all three of the following:
	*Serum aminotransferases that are >2 × ULN on ≥2 occasions
	AND
	*CBD dilation ≥10 mm on US or 12 mm on ERCP
	AND
	*Delayed drainage (>45 min) of contrast from the CBD on ERCP
**Type 2**	Biliary pain associated with one or two of the following:
	*Serum aminotransferases that are >2 × ULN on ≥2 occasions
	OR
	*CBD dilation ≥10 mm on US or 12 mm on ERCP
	OR
	*Delayed drainage (>45 min) of contrast from the CBD on ERCP
**Type 3**	Biliary pain only

*CBD, common bile duct; ERCP, endoscopic retrograde cholangiopancreatography; SOD, sphincter of Oddi dysfunction; ULN, upper limit of normal; US, ultrasonography*.

**Table 2:** **Milwaukee classification for pancreatic SOD (39)**.

**Type 1**	Pancreatic type pain associated with all three of the following:
	*Serum amylase or lipase that is >2 × ULN on ≥2 occasions
	AND
	*Pancreatic duct (PD) dilation >6 mm in head and >5 mm in body
	AND
	*Delayed drainage (>9 min) of contrast from the PD on ERCP
**Type 2**	Pancreatic type pain associated with one or two of the following:
	*Serum amylase or lipase that is >2 × ULN on ≥2 occasions
	OR
	*PD dilation >6 mm in head and >5 mm in body
	OR
	*Delayed drainage (>9 min) of contrast from the PD on ERCP
**Type 3**	Pancreatic type pain only

*ERCP, endoscopic retrograde cholangiopancreatography; SOD, sphincter of Oddi dysfunction; ULN, upper limit of normal*.

**Table 3:** **Rome III classification of SOD**.

Epigastric or right upper quadrant abdominal pain that is associated with ≥1 of the following:
≥30 min in duration
Recurrent symptoms occurring at variable intervals (not daily)
Occurring on ≥1 occasion in the past 12 months
Pain that builds up to a steady level
Pain that is moderate to severe enough to interrupt daily activity
No evidence of structural abnormalities

*SOD, sphincter of Oddi dysfunction*.

## Other Evaluations for SOD

In the past, non-invasive testing to diagnose SOD included quantitative hepatobiliary scintigraphy to assess biliary flow (42–44), endoscopic ultrasound, or magnetic resonance cholangiopancreatography with secretin injection (45). However, these tests are neither sensitive nor specific for SOD.

Manometry of the SO is considered the gold-standard test to diagnose SOD (46, 47). During SO manometry, a catheter is inserted into the duodenum and calibrated to 0 mmHg. Next, the catheter is inserted into the CBD and/or PD for 30 s; basal pressures ≥40 mmHg indicate SOD (48). Prior to the procedure, patients should avoid agents that inhibit SO function, such as anticholinergics, nitrates, and calcium-channel blockers, and those that stimulate it, such as opiates and cholinergics. There are limitations to SO manometry: first, it requires skilled endoscopists with special equipment not readily available at most institutions; second, it is associated with up to 30% increased risk of iatrogenic pancreatitis (49); and finally, isolated point in time pressure measurements obtained during SO manometry may not reflect the dynamic nature of the SO, leading to difficulties in applying the results. Therefore, the use of SO manometry as a gold-standard test remains controversial. Furthermore, isolated basal pressures cannot differentiate between SO motor disturbances and anatomical stenosis. Of note, manometry is not confirmatory in 13–40% of patients ultimately diagnosed with type 1 SOD (50–53).

## Non-Pharmacologic Risk Factors for SOD

Certain populations, such as patients who have undergone cholecystectomy (32), are predisposed to SOD (31). In subjects with an intact gallbladder, CCK inhibits SO phasic wave activity, but 6 months after cholecystectomy, CCK fails to inhibit this activity (54). Sherman reported that 10–20% of postcholecystectomy patients experience biliary colic and a review of patients with postcholecystectomy pain found that 9–51% met diagnostic criteria for SOD after cholecystectomy (31). Overall, ~1.5% of patients develop SOD after cholecystectomy (25, 55).

It is postulated that the gallbladder acts as a backflow reservoir for bile to dampen sudden increases in pressure resulting from physiologic or extra-physiologic ductal obstruction (56–59). Luman and colleagues demonstrated that patients with postcholecystectomy syndrome had elevated basal SO pressure, retrograde phasic wave contraction, and an increase in phasic wave frequency greater than seven contractions per minute (tachyoddia) (60). It is unclear if postcholecystectomy patients are susceptible to developing SOD because of elevated pressures, altered SO motility, or both.

Sphincter of Oddi dysfunction has also been linked to agenesis of the gallbladder (61), preoperative cholelithiasis (25), gallstone lithotripsy (62), liver transplantation (63), and alcoholism (64). Delayed emptying of the biliary tract related to hypothyroidism suggests another risk factor for SOD (65, 66). Additionally, subjects with hypothyroidism have an increased risk of gallstones, thought secondary to the absence of thyroxine’s relaxing effect on the SO (67).

Patients with irritable bowel syndrome (IBS) may be at an increased risk of SOD. Evans and colleagues reported that patients with IBS who undergo cholecystectomy are more likely to demonstrate a blunted response to sphincter-relaxing properties of CCK compared with postcholecystectomy patients without IBS (68). The prevalence and incidence of SOD among IBS patients is unclear because of the difficulty in diagnosing SOD in IBS patients due to overlap of symptoms (69).

Sphincter of Oddi dyskinesia occurs more frequently in women than in men, and animal models offer some insight. In the prairie dog, CCK increases SO phasic wave frequency in both sexes, but amplitude increases were significantly greater in females than in males (70). Further support for gender differences was shown by estrogen suppression of SO wave frequency in prairie dogs, while progesterone’s effect on the SO is unclear (71).

## Medications and the Risk of SOD

Exogenous agents play an additive role in populations at risk for SOD. Opiates are known to alter flow through the SO. In the absence of a gallbladder, morphine, meperidine, and pentazocine increase biliary pressure in opossums (56). Behar and Biancani found that leucine and methionine-enkephalin caused an initial contraction followed by a prolonged relaxation of the cat SO, suggesting that endogenous delta opioid agonism is involved in increasing flow through the sphincter (72, 73). Importantly, the mu antagonist naloxone had no effect on morphine-induced increases in basal SO pressure but did diminish morphine’s effect on SO phasic wave frequency and amplitude. The naloxone inhibitory effect suggests that the mu opioid receptor is involved, while the absence of naloxone antagonism on SO basal pressure may be non-mu opioid receptor mediated.

Thus, morphine increases the amplitude and frequency of the phasic wave (*via* mu opioid receptors) as well as basal pressure (*via* non-mu opioid receptors) of the SO (74, 75). These effects have also been demonstrated with fentanyl (76) and codeine (77). Morphine shows limited effect on the SO in patients prior to cholecystectomy, whereas it caused a notable rise in basal sphincter pressure postoperatively (59). Since morphine can precipitate abdominal pain not related to the SO, Holtzer and Hulst proposed a “morphine-enzyme-pain” provocation test: pain accompanied by at least a doubling of the alanine aminotransferase 8 h after administration indicated SO dyskinesia (75). The magnitude of transaminase elevation associated with morphine has been reported as high as 65 times above normal in patients without a gallbladder (78). Mousavi and colleagues demonstrated that chronic opiates induce SOD compared with case controls (79), and several authors have documented asymptomatic, dilated CBDs in patients addicted to opiates (80–82).

The effects of tramadol, buprenorphine, pentazocine, and pethidine have been evaluated with SO manometry. Pentazocine increased the duration of sphincter contraction and ductal pressure while tramadol, buprenorphine, and pethidine did not (83, 84). Meperidine was shown to increase the pancreatic component, biliary component, and SO phasic frequency and to decrease phasic duration in 3/47 patients studied with manometry (85). Eluxadoline, a mixed opioid receptor modulator with mu and kappa opioid receptor agonist effects and delta opioid receptor antagonist effects that was recently approved by the FDA for IBS with diarrhea, was linked to a small number of non-serious cases of SO spasm and pancreatitis in the phase 3 studies of this medication (86). Among 1,619 patients exposed to eluxadoline in these trials, 8 (0.49%) developed SO spasm and 5 (0.31%) developed pancreatitis, 1 case of which was attributed to SO spasm. Importantly, all cases of eluxadoline-associated SO spasm occurred in patients who did not have gallbladders and were more common with 100 mg twice daily (BID) compared with 75 mg BID. One case of pancreatitis was associated with biliary sludge while the other three were associated with heavy alcohol use.

Opiates have been reported to incite pancreatitis, and their effects on the SO represent the most likely etiology (87). The mu antagonist naloxone reduces the severity of pancreatitis induced by intraductal injection of trypsin–bile mixture in dogs. In the opossum, Chen and colleagues induced pancreatitis when they combined simulated SOD (by PD ligation mimicking the opiate effect) with pharmacologically stimulated pancreatic secretion (88). In humans, drug rechallenge (89, 90) with heroin (91, 92), codeine (93–95), tapentadol,^1^ and loperamide (96–99) have established a link with these drugs and acute pancreatitis. Loperamide inhibits the normal contractile response of the gallbladder to CCK (100) and, in patients with short bowel syndrome, reduces pancreatic and biliary output (101). The mechanism of opiate-induced pancreatitis is assumed to be related to their action on the SO; some suggest that loperamide’s effect may be different as it is structurally more related to haloperidol (102) than to morphine.

## Therapy for SOD

Certain exogenous agents relax the SO, reducing its pressure and resistance. This includes calcium-channel blockers (103, 104), tricyclic antidepressants (105), Botox^®^, glyceryl trinitrate (GTN), and somatostatin. Nifedipine has been shown to reverse opiate-induced effects on the SO (106) and improve pain associated with SOD in a short-term study (103). Injection of Botox^®^ into the SO *via* sclerotherapy needle reduced sphincter pressure by 50% for 4 months and was followed by 50% improvement in bile flow in two patients with postcholecystectomy pain and elevated SO pressures (107). GTN has been used to assist removal of lodged CBD stones without endoscopic papillary dilatation or endoscopic papillotomy (108) and decreased both basal SO pressure as well as the amplitude and frequency of SO phasic wave contractions in a non-randomized, controlled clinical trial (108). Intravenous somatostatin was shown to reduce mean SO (109) basal pressures in patients with acute alcoholic pancreatitis (64).

One prospective study of patients with biliary SOD (defined by clinical and laboratory data) evaluated the combination of a low-dose tricyclic antidepressants, nifedipine, and GTN. If there was no improvement after 3–6 months, patients were offered biliary sphincterotomy. Fifty-one percent of the patients (76% with type 3) had symptomatic resolution or improvement on medical therapy alone, 12% had symptomatic improvement or resolution with sphincterotomy, and 10% had improvement with both medical therapy and sphincterotomy (110). Although promising, opiates were allowed during this study, confounding the determination of symptom improvement due solely to interventions.

The most commonly used non-pharmacologic treatment for SOD has been endoscopic sphincterotomy for patients with types 1 and 2 biliary SOD and pancreatic SOD. Pain relief has been shown in 90% of patients with type 1 biliary SOD and 70% of patients with type 2. However, it is not effective, and may be harmful, in patients with type 3. The evaluating predictors and interventions in sphincter of Oddi dysfunction trial was a landmark study for treatment of type 3. This was a multicenter, sham-controlled, randomized trial in patients with pain after cholecystectomy, without abnormalities on imaging or laboratory studies, and no prior SO treatment. Participants underwent sphincterotomy or sham sphincterotomy for abdominal pain. The investigators concluded that performing a sphincterotomy in patients with type 3 SOD was ineffective (111). As a result, endoscopists are shifting away from performing sphincterotomy in these patients.

There are limited studies evaluating the role of PD stenting and sphincterotomy in patients with pancreatic SOD. Jacob and colleagues found a significant reduction in the incidence of recurrent acute pancreatitis in those who were stented (112). Coté and colleagues evaluated the role of endoscopic dual (biliary and pancreatic) sphincterotomy vs. biliary sphincterotomy alone in patients with IARP and found that both types of sphincterotomies had similar effects in preventing recurrence of acute pancreatitis (113). Another study found no difference in preventing recurrent pancreatitis when dual sphincterotomy was compared with either pancreatic or biliary sphincterotomy (114).

## Conclusion

Sphincter of Oddi dysfunction denotes impaired fluid flow through the SO, either by a fixed stenosis or disordered muscular control (dyskinesia). Gallbladder function appears to play a critical role in SO mechanics, and patients without a gallbladder are more likely to experience SOD. Other potential contributing factors include female gender, hypothyroidism, IBS, prior pancreatitis, and exogenous medications. The data supporting a link between opiates and SOD are clear and reproducible, and the resulting clinical syndromes, especially in postcholecystectomy patients, include abdominal pain with sudden, yet reversible, elevations in liver enzymes as well as acute pancreatitis. Different opiate agents appear to have varying effects on SO basal and phasic contractions. While exogenous mu opioid agonists negatively affect flow through the SO, endogenous enkephalins (possibly delta agonists) may improve flow through the sphincter.

While there is some evidence that nifedipine, Botox^®^, and GTN may improve flow through the SO and mitigate SOD symptoms, the efficacy of these agents remains to be proven in sufficiently large, randomized, and controlled trials. Endoscopic sphincterotomy remains the treatment of choice for select patients confidently diagnosed with SOD. However, increased awareness by caregivers of risk factors for SOD provides opportunities for diagnosis and intervention, including avoidance of potential precipitating agents, especially in the absence of a gallbladder.

## Author Contributions

EA, SP, and PC were involved in the initial drafting of the manuscript. EA, SL, PC, BC, and SP were involved in the drafting and revision of the manuscript. All authors approved the final draft of this manuscript for submission. SP is the guarantor of the article.

## Conflict of Interest Statement

PC serves as a scientific consultant for Allergan plc. BC has served as an advisor, consultant, or speaker for Actavis, Inc., a subsidiary of Allergan plc, Salix Pharmaceuticals, Takeda Pharmaceuticals, Prometheus Laboratories, IM HealthScience, and Ironwood Pharmaceuticals. SP serves as a member of the VIBERZI Response Team for Allergan plc, monitoring use and adverse responses, and is the Specialty Chief Editor for the Gastrointestinal Sciences section of *Frontiers*. EA and SL have no relevant disclosures to report.

## Footnotes

^1^http://www.accessdata.fda.gov/drugsatfda_docs/nda/2008/022304s000_MedR_P2.pdf.
